# Effects of blended learning on physical fitness and basketball skills of male college students: a cluster randomized controlled trial

**DOI:** 10.3389/fpsyg.2026.1792646

**Published:** 2026-03-31

**Authors:** Zexu Li, Xutao Liu

**Affiliations:** School of Physical Education, Jiangsu University of Science and Technology, Zhenjiang, China

**Keywords:** basketball skills, blended learning, higher education, physical fitness, psychology

## Abstract

**Background:**

University represents a critical period for the cultivation of health behaviors, self-efficacy, and psychosocial development. It affects how well students do in their studies, their day-to-day life, and where they're headed in the future. However, in recent years, the physical fitness levels of Chinese university students have declined. This study investigated, through a 12-week intervention, whether a blended learning method could improve the physical fitness and basketball skills of male university students in China.

**Methods:**

A total of 69 male first-year students from Jiangsu University of Science and Technology (mean age = 18.6 ± 0.7 years; range = 18–20 years) were recruited and randomly assigned to either an experimental group (blended learning) or a control group (traditional learning) using a cluster randomized controlled trial design. The experimental group engaged in a blended learning approach, which integrated several key components. The approach included pre-class MOOC-based instructional videos, in-class differentiated skill training, and post-class online quizzes with reflective tasks. Meanwhile, the control group followed a traditional teacher-centered model. Fitness measures included body mass index (BMI), vital capacity, pull-ups, standing long jump, 50-meter sprint, and 1,000-meter run, while basketball skills were evaluated through set shot and half-court dribbling-and-shooting test. All data were analyzed with SPSS 27.0, with the significance level set at *p* < 0.05.

**Results:**

Following the 12-week intervention, both learning approaches significantly enhanced overall physical fitness. Significant differences between groups favoring the experimental group were observed in pull-ups (*p* < 0.001, d = 2.03) and standing long jump (*p* < 0.001, d = 1.04). In contrast, no significant difference was found for the 1,000-meter run (*p* = 0.597). Notably, the experimental group showed a greater reduction in BMI (d = −0.54) and greater improvement in 50-meter sprint times (d = −0.30) compared to the control group. In basketball skills, the experimental group surpassed the control group with large effect sizes for both the set shot (d = 1.63) and half-court dribbling and shooting (d = 1.55).

**Conclusion:**

The 12-week blended learning approach enhanced the overall physical fitness of university students. Furthermore, this approach was highly effective in promoting the development of basketball skills. However, the approach showed limited effects on BMI and sprint outcomes, suggesting that blended learning may require enhanced explosive training components to address these fitness dimensions.

## 1 Introduction

Higher education plays a key role in establishing lifelong health behaviors, self-efficacy, and psychosocial development. Physical fitness during this period is closely linked to academic achievement, quality of life, and broader societal outcomes (Redondo-Flórez et al., 2022). Research indicates that high-quality physical education courses not only enhance students' physical fitness and motor skills, but also cultivate positive exercise attitudes, social skills, emotional management (e.g., stress reduction), and social responsibility through teamwork and community involvement (Li et al., 2014; Peltzer et al., 2014; Fan and Yu, 2017; Goudas and Magotsiou, 2009). In recent years, a downward trend in the physical fitness levels of university students has been observed, marked by a concurrent rise in the prevalence of overweight individuals (Peltzer et al., 2014). However, physical education in many Chinese universities is constrained by systemic limitations, including an insufficient number of qualified teachers, a lack of diversified instructional resources, and a continued reliance on traditional, single-mode teaching methods (Fan and Yu, 2017). These constraints hinder overall teaching effectiveness and are associated with declining student fitness levels, particularly in male first-year cohorts (Dong et al., 2023). Such physical decline is further linked to a growing burden of cardiovascular risks, including being overweight, obesity, and elevated blood pressure among college students (Tran et al., 2017). These issues adversely affect students' physical and mental health, academic performance, and their long-term wellbeing. In response, educators and researchers are actively seeking more effective and flexible instructional approaches to provide engaging learning experiences, improve fitness outcomes, and enhance motor skills in children. Physical education is a basic way to improve students' awareness of exercise, physical activity, and physical fitness (Mandrillon et al., 2024). However, with the advancement of society and information technology, physical education in Chinese universities now faces many challenges (Wang, 2017). Most universities still use traditional teaching methods to teach students. This teacher-centered approach makes students learn passively, reduces their interest in sports, and lowers their motivation to exercise independently outside of class (Alimohammadi et al., 2017). Simultaneously, traditional teaching ignores students' individual differences. With limited class time, teachers find it difficult to teach students according to their needs (Colquitt et al., 2017). In addition, this approach depends too much on teachers' ability and limited teaching resources; therefore, it cannot meet the diverse learning needs of today's students. Another problem is the evaluation system, which is often too simple, ignores the importance of assessing the learning process, and fails to fully and fairly reflect students' progress (Hernán et al., 2023). Research shows that using effective teaching methods can significantly improve college students' fitness, including their BMI, lung capacity, speed, and endurance (Yuksel et al., 2020; Troosters et al., 2023; Ardoy et al., 2011).

Advanced Internet technologies have powerfully driven the development of blended learning in higher education settings. Tools such as Learning Management Systems (LMS), online video platforms, and interactive digital tools have opened up new possibilities for pedagogical innovation (Nasir et al., 2021; Bhadri and Patil, 2022). Blended learning is an innovative instructional approach that combines online and traditional, in-person teaching. Recently, it has gained significant recognition in education and continues to attract increasing interest (Adams, 2013). Blended learning overcomes the limitations of traditional teaching by using the key benefits of online learning: flexibility in time and place, easy sharing of resources, and quick feedback (Saltan, 2017). Research shows that changing from traditional teaching to student-focused blended learning helps students develop advanced thinking skills and improves how they learn and use knowledge (Caulfield, 2023). This change in teaching also leads to classes that are more interactive and interesting, helping students achieve better results (Jou et al., 2016).

While blended learning has been successfully integrated into many higher education courses in China (Castro, 2019), research remains predominantly focused on subjects such as English, computer science, accounting, and mathematics (Tomlinson and Whittaker, 2013; Pérez-Marín and Pascual-Nieto, 2012; Borba et al., 2016). However, existing studies on blended learning in physical education have mainly focused on subjective outcomes such as motivation, engagement, and attitudes (Bayyat, 2020; Huy and Vu, 2020; Moon and Park, 2023), with limited use of randomized controlled designs and objective physical fitness measurements. Moreover, basketball-specific skill acquisition within a cluster randomized controlled trial (CRCT) framework remains underexplored, despite the sport's popularity and technical complexity. In contrast, basketball is exceptionally popular among Chinese university students (Wang et al., 2024a), and enjoys widespread participation and appeal, specifically among male students at Jiangsu University of Science and Technology. Its unique technical and tactical demands suggest that it may yield distinct learning outcomes within a blended approach.

Basketball was chosen for the intervention because it is a good fit for testing blended learning. It is very popular among Chinese male university students, which helps keep them engaged and motivated (Wang et al., 2024a). Also, the sport is quite complex. It involves different skills like shooting, dribbling, and passing, as well as quick decision-making. That means players naturally need a lot of practice and feedback, which fits well with the blended learning approach. Lastly, basketball also works on different parts of fitness, such as strength, explosive power, and coordination. So, it gives us a good way to see how well the intervention works for both skill learning and physical development.

Therefore, this study aimed to investigate the effects of a blended learning approach on the physical fitness and basketball skills of male university students at Jiangsu University of Science and Technology. We predicted that, compared to traditional teaching methods, the blended learning model would lead to significantly greater improvements in both fitness indicators and sport-specific skill performance.

## 2 Materials and methods

### 2.1 Participants

This study was designed as a cluster randomized controlled trial (CRCT) following the CONSORT statement. The required sample size was determined a priori using G-Power version 3.1 (Kang, 2021). The effect size was set at 0.20 based on the previous literature. With α = 0.05 and power (1-β) = 0.80, the minimum sample size was determined to be 52. Accounting for the design effect of cluster randomization and an anticipated dropout rate of 20%, the total sample size was adjusted to 60 students, with 30 students in each group.

To ensure sufficient statistical power after potential attrition, a total of 70 male volunteers were recruited from two intact first-year basketball classes at Jiangsu University of Science and Technology. To ensure sample homogeneity, the exclusion criteria were as follows: (1) any pre-existing medical condition or long-term medication use affecting participation (e.g., cardiovascular, respiratory, or musculoskeletal issues); (2) prior experience (within the last year) with similar blended learning approaches or structured physical training/basketball practice; and (3) voluntary withdrawal without providing a reason during the trial.

**Figure 1:**
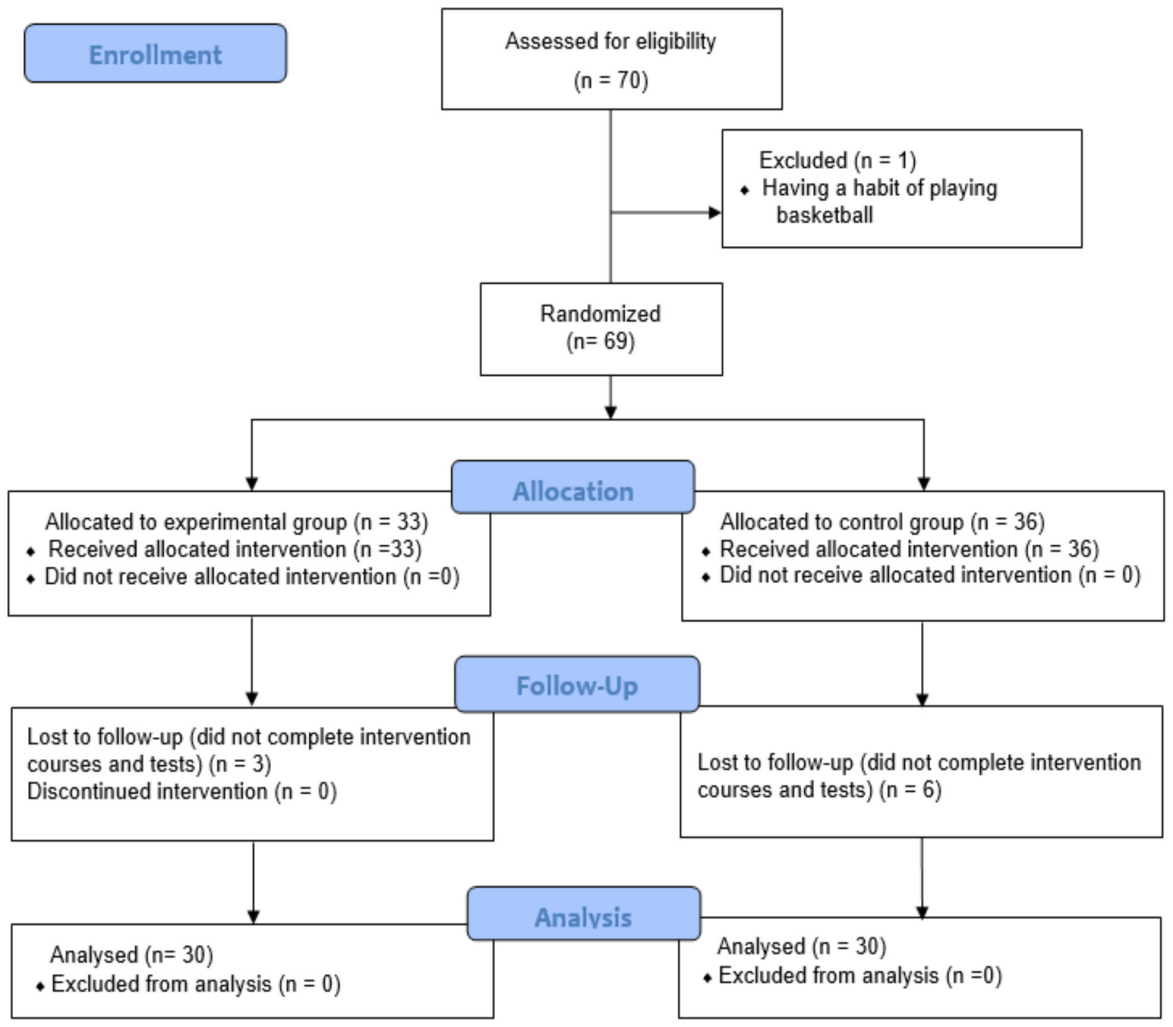
CONSORT flow diagram.

The study steps are illustrated in Figure 1. Using a cluster randomized controlled trial design, the two intact classes were randomly allocated to the experimental group (blended learning) or the control group (traditional learning) by an independent researcher using a computer-generated random number sequence. After screening, 69 eligible students were included, with 33 in the experimental group and 36 in the control group. During the 12-week intervention, three participants in the experimental group and six in the control group dropped out (reasons: injury unrelated to the study, scheduling conflicts, and personal withdrawal). Thus, the final analysis included 30 participants in each group (see CONSORT flow diagram, Figure 1).

Table 1 shows the baseline characteristics of the participants. The mean age was 18.6 years (SD = 0.7), mean height was 175.3 cm (SD = 5.2), mean weight was 68.4 kg (SD = 7.1), and mean BMI was 22.3 kg/m^2^ (SD = 2.1). Independent *t*-tests showed no significant differences between the groups at baseline for any variable (all *p* > 0.05). All participants signed a consent form to participate, and the study was approved by the Ethics Committee of Jiangsu University of Science and Technology (Approval No. JUST-2025-015).

**Table 1:** Baseline characteristics of participants.

Variable	Experimental group (*n* = 30)	Control group (*n* = 30)	*p*
Age (years)	18.5 ± 0.6	18.7 ± 0.7	0.324
Height (cm)	175.1 ± 5.0	175.5 ± 5.4	0.768
Weight (kg)	68.1 ± 6.8	68.7 ± 7.4	0.712
BMI (kg/m^2^)	22.2 ± 2.0	22.4 ± 2.2	0.691

Values are mean ± SD. Independent t-tests showed no significant differences between groups.

### 2.2 Intervention

To avoid interference between the two teaching approaches, the experimental and control groups were scheduled at different times (8:30–10:05 a.m. for the experimental group, and 10:20–11:55 a.m. for the control group) and used different basketball courts. The two groups did not interact during the study. The same lecturer taught both groups to keep the content and intensity consistent (Table 2). Both groups followed three main phases: pre-, during, and post-class. The in-class instruction consisted of a 20-min preparation session, a 60-min core session, and a 10-min concluding session (see Table 3). The control group received instruction through a conventional teacher-centered approach, relying primarily on in-class explanations and demonstrations, with pre- and post-class study left to students' self-direction. The experimental group followed a blended learning format. Before class, the experimental group watched short videos (under 10 min each, recorded by experienced coaches) on the widely used Chinese University MOOC platform (https://www.icourse163.org). The platform continuously recorded students' viewing duration, quiz completion, and forum activity, enabling the lecturer to track engagement and offer timely feedback. This provided a flexible and convenient way to preview the material.

**Table 2:** Basketball teaching plan.

Week	Teaching content	Teaching methods (TL)	Teaching methods (BL)
1	Basketball basic knowledge, athletic preparation stance, high-intensity stationary ball control drills	Explanation Demonstration	Online video explanation and demonstration Online quiz
2	Offensive movement techniques (explosive start, crossover breakthrough footwork, quick direction change)	Explanation Demonstration	Online video explanation and demonstration Online quiz
3	Defensive movement techniques (lateral slide, pursuit and retreat step, closeout footwork) Physical fitness: Sprint 30 m × 5 sets, Push-ups 20 reps × 3 sets	Explanation Demonstration	Online video explanation and demonstration Online quiz
4	Passing skills (one-handed chest pass, bounce pass, overhead pass) Physical fitness: Squats 25 reps × 3 sets, Pull-ups 10 reps × 2 sets	Explanation Demonstration	Online video explanation and demonstration Online quiz
5	Dribbling skills (stationary dribble, variable speed/variable direction dribble, behind-the-back dribble intro) Physical fitness: Vertical Jump 25 reps × 3 sets, 400 m Sprint × 2 sets	Explanation Demonstration Group competitive drills	Online video explanation and demonstration Group competitive drills Online test
6	Shooting Skills (Standing One-handed Shoulder Shot, Catch-and-Shoot Jump Shot) Physical Fitness: Plank 90s × 3 sets, Push-ups 18 reps × 3 sets	Explanation Demonstration Group competitive drills	Online video explanation and demonstration Group competitive drills Online test
7	Dynamic shooting and layup (running one-handed shoulder shot, three-step layup intensification) Physical Fitness: Half-court shuttle run × 3 sets, 1-minute Jump Rope × 4 sets	Explanation Demonstration Group competitive drills	Online video explanation and demonstration Group competitive drills Online test
8	Advanced layup skills (running one-handed underhand layup, reverse layup, floater) Physical fitness: Burpees 15 reps × 3 sets, Sprint 60m × 4 sets	Explanation Demonstration Group competitive drills	Online video explanation and demonstration Group competitive drills Online test
9	Combined Skills (jump shot + crossover breakthrough, dribble-drive and kickout pass) Physical fitness: Pull-ups 12 reps × 3 sets, Box Jumps 20 reps × 3 sets	Explanation Demonstration Group competitive drills	Online video explanation and demonstration Group competitive drills Online test
10	Breakthrough Techniques (Crossover Step Breakthrough, Same-Side Step Breakthrough, Spin Move Breakthrough) Physical Fitness: 300m Interval Run × 3 sets, Squats 30 reps × 3 sets	Explanation Demonstration Group competitive drills	Online video explanation and demonstration Group competitive drills Online test
11	Full-court fast break and transition offense Physical fitness: 1-min Jump Rope × 5 sets, Push-ups 22 reps × 3 sets Half-court 3V3 teaching competition	Explanation Demonstration Group competitive drills	Online Video explanation and demonstration Group competitive drills Online test
12	Comprehensive skill and tactics review Physical fitness test (speed: 50 m sprint; explosiveness: vertical jump; muscular endurance: pull-ups/push-ups; aerobic capacity: 1,000 m Run) Full-court 4V4 teaching competition	Explanation Demonstration Group competitive drills	Online video review and guidance Online comprehensive test

**Table 3:** Basketball teaching implementation.

Time period	Control group (traditional teaching)	Experimental group (blended teaching)
Pre-class (5–10 min)	1. Preview new technical points (e.g., breakthrough footwork, dynamic shooting) 2. Review key points of previous skills	1. Watch targeted online demonstration videos (focus on male-oriented explosive movements) 2. Complete online quiz on technical essentials 3. Submit pre-class practice feedback
During class (90 min)	Preparation Section (20 min) 1. Procedures (Attendance check + Clarify learning objectives + Safety tips for confrontational training) 2. High-intensity warm-up (sprint shuttle, dynamic stretching, joint activation)	Preparation Section (20 min) 1. Procedures (Attendance check + Analyze pre-class quiz results + Emphasize difficult points) 2. Customized warm-up (combined with online video guidance, focusing on lower limb explosion and core stability)
	Basic Section (60 min) 1. Review previous skills (group rotation practice) 2. Explain and demonstrate new skills (e.g., crossover breakthrough, reverse layup) 3. Individual skill polishing + small-group drill (2V2) 4. Physical fitness training (pull-ups, 30m sprint, box jumps)	Basic Section (60 min) 1. Review of online video playback (targeted correction of pre-class feedback problems) 2. Demonstrate and explain new skills (focus on solving common mistakes in male confrontational scenarios) 3. Confrontational group practice (3V3) + real-time guidance 4. Intensive physical training (interval running, weighted squats, plank with shoulder taps)
	Conclusion Section (10 min) 1. Relaxation stretch (focus on muscle groups used in training) 2. Class summary + assignment arrangement (offline skill repetition)	Conclusion Section (10 min) 1. Guided relaxation (combined with online audio instructions) 2. Summary of confrontational performance + personalized improvement suggestions 3. Assign online reflection task (record practice video for teacher review)
After-class (1–3 min)	1. Consolidate key skills through offline practice 2. Complete online knowledge test	1. Reflect on confrontational performance and summarize improvement points 2. Upload practice video to online platform 3. Participate in peer skill evaluation

Additionally, the MOOC platform's discussion forum allowed students to exchange questions and clarify technical challenges from the videos with their classmates and the instructor, thereby promoting deeper engagement and comprehension of the material. As students had already acquired a basic understanding of the content before class, more in-class time in the experimental group could be allocated to practicing skills and improving physical fitness, which enabled the teacher to provide tailored guidance to a greater number of students.

After class, students in the experimental group took a short online quiz that was automatically graded. The quiz had six multiple-choice questions to help remember the main lessons, and it immediately showed them the correct answers and explanations so they could fix any mistakes quickly. The group also used a WeChat chat where the teacher sent learning videos and task reminders 2 days before class, so students could ask questions and get help. Records of their online learning and attendance were kept track of how much they took part. Everyone was asked to maintain a regular daily schedule and inform the teacher if their exercise habits changed.

### 2.3 Evaluation

Teaching effectiveness was evaluated using a double-blind method by experienced fitness professionals. All tests closely followed the “National Student Physical Health Standard (2014 Revision)” (see Table 4). Published by China's Ministry of Education, these standards measure students' physical fitness in three areas: body shape, body function, and physical ability, including strength, speed, endurance, flexibility, and agility. The NSPHS (2014 Revision) is a well-tested and widely used fitness assessment tool in Chinese universities (Mao et al., 2022; Deng and Fitzpatrick, 2025).

**Table 4:** National student physical health standard (2014).

Score	BMI	Lung capacity (ml)	50-meter run (s)	Standing long jump (cm)	1-min pull-up (number of times)	1,000-meter run (m·s)
	M	M	M	M	M	M
100	17.9–23.9	5,040	6.7	19	56	3′17″
95		4,920	6.8	18	54	3′22″
90		4,800	6.9	17	52	3′27″
85		4,550	7.0	16	49	3′34″
80	≤ 17.8; 24.0–27.9	4,300	7.1	15	46	3′42″
78		4,180	7.3		44	3′47″
76		4,060	7.5	14	42	3′52″
74		3,940	7.7		40	3′57″
72		3,820	7.9	13	38	4′02″
70		3,700	8.1		36	4′07″
68		3,580	8.3	12	34	4′12″
66		3,460	8.5		32	4′17″
64		3,340	8.7	11	30	4′22″
62		3,220	8.9		28	4′27″
60	≥28.0	3,100	9.1	10	26	4′32″
50		2,940	9.3	9	24	4′52″
40		2,780	9.5	8	22	5′12″
30		2,620	9.7	7	20	5′32″
20		2,460	9.9	6	18	5′52″
10		2,300	10.1	5	16	6′12″

M, male.

Body shape was assessed using body mass index (BMI), calculated as weight (kg) divided by height squared (m^2^). Physical function was measured via lung capacity. Physical fitness was evaluated through five tests: 50-meter run, standing long jump, sit-and-reach, pull-ups, and 1,000-meter run. All participants were tested before and after the intervention by the same evaluator, following the fixed order listed above and using standardized instruments from the National Student Physical Health Test. Each test item was scored on a 100-point scale.

**Figure 2:**
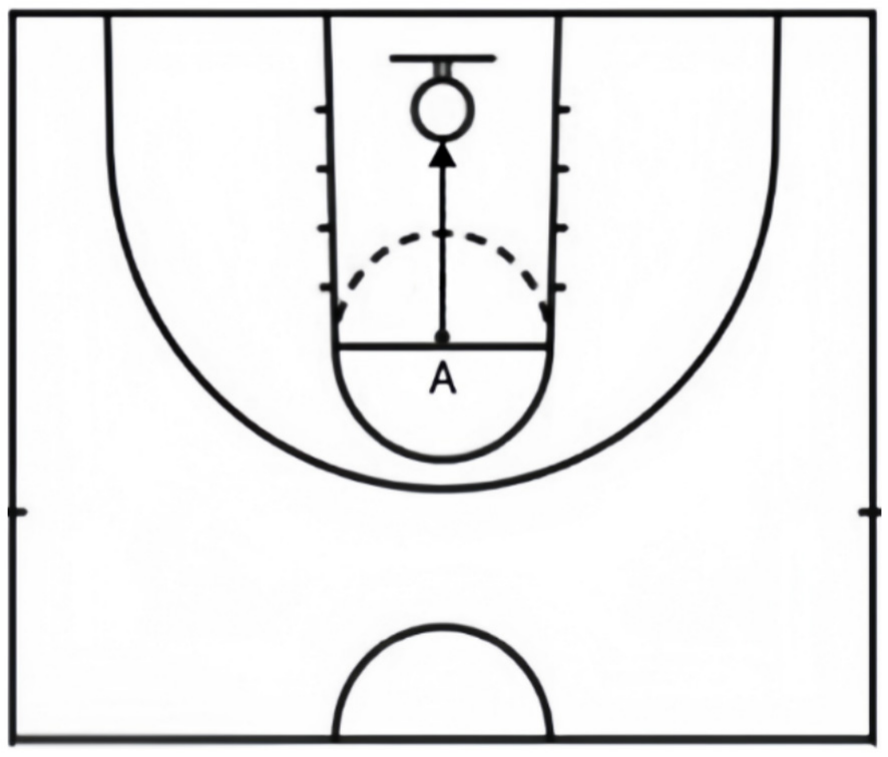
Set shot.

**Figure 3:**
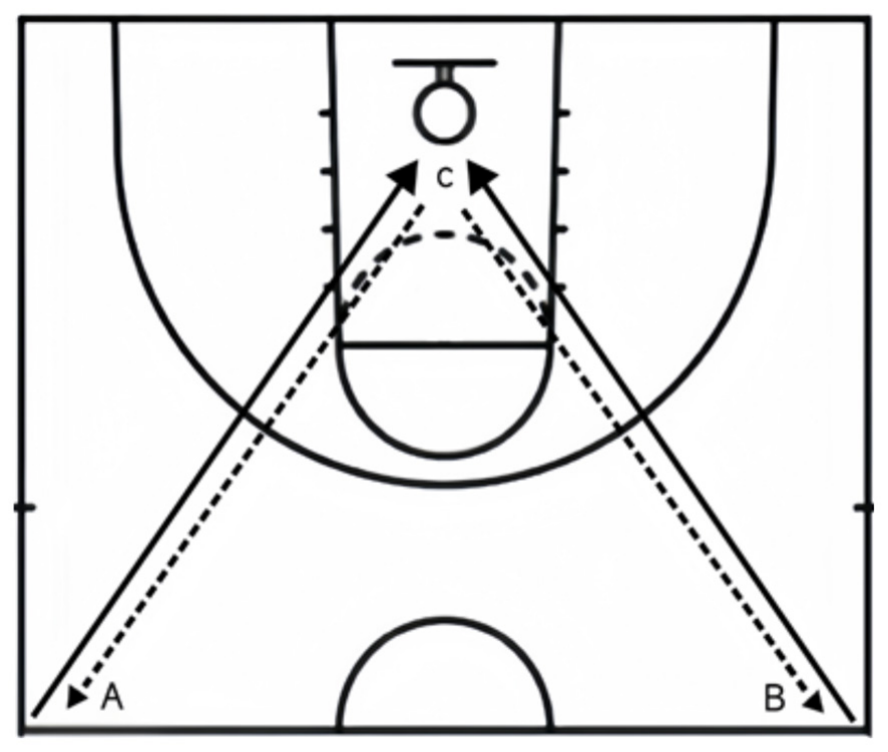
Half-court dribbling and shooting.

Basketball skills were assessed using two tests widely adopted in Chinese college settings: the set shot test and the half-court dribbling and shooting test. In the set shot test, each student took ten attempts from the free-throw line (point A), with the referee recording the number of successful shots and a technical score (see Figure 2). In the half-court dribbling and shooting test (see Figure 3), the student began at point B facing the basket, dribbled with the right hand to point C for a shot, continued to point A, switched to the left hand, dribbled back to point C for another shot, and finally returned to point B—with the timer stopping upon return. Each participant performed two trials, and the highest score and technical score were recorded. The tests were evaluated by six experts in physical education and exercise training, showing excellent content validity (I-CVI = 0.833–1.000, Kappa = 0.816–1.000).

Inter-rater agreement for the basketball skill assessments was evaluated using a two-way random effects model. The intraclass correlation coefficient (ICC) was 0.91 [95% CI [0.86, 0.94]] for the set shot test and 0.89 [95% CI [0.83, 0.93]] for the half-court dribbling and shooting test, indicating excellent consistency.

### 2.4 Statistics

All statistical analyses were performed using SPSS 27.0. Normality was checked with Shapiro–Wilk tests, and equality of variances with Levene's test. Independent *t*-tests were used to compare baseline data between the two groups, and no significant differences were found. Paired t-tests were used to examine changes from pre-test to post-test within each group. Independent *t*-tests were also used to compare the two groups at post-test. Cohen's d was calculated. For within-group changes, we divided the mean difference by the SD of the difference. For between-group comparisons, we divided the mean difference by the pooled SD. Effect sizes were interpreted as 0.2 = small, 0.5 = medium, and 0.8 = large (Durlak, 2009). Statistical significance was set at *p* < 0.05.

## 3 Results

### 3.1 Physical fitness outcomes

The effects of the blended learning approach on university students' physical fitness after the 12-week intervention are shown in Table 5. Overall, the blended learning approach led to significant post-intervention improvements in lung capacity (*p* < 0.001, d = 1.14), standing long jump (*p* < 0.001, d = 1.04), and pull-ups (*p* < 0.001, d = 2.03). In contrast, BMI (d = −0.54) and 50-meter sprint (d = −0.30) exhibited negative effect sizes, indicating that the experimental group showed less favorable changes on these measures compared to the control group. For the 1,000-meter run, no significant between-group difference was found (*p* = 0.597).

**Table 5:** Physical fitness outcomes.

Outcome	Group	Pre-test (M ±SD)	Post-test (M ±SD)	Within-group *p*^a^	Between-group *p*^b^	Cohen's d^c^
BMI (kg/m^2^)	EG	21.99 ± 2.21	21.22 ± 2.20	< 0.001	0.043	−0.54
	CG	22.82 ± 2.35	22.45 ± 2.39	0.002		
Lung capacity (ml)	EG	3629.17 ± 421.61	4006.50 ± 422.64	< 0.001	< 0.001	1.14
	CG	3696.73 ± 438.22	3765.37 ± 444.91	< 0.001		
50-m sprint (s)	EG	7.60 ± 0.49	7.19 ± 0.48	< 0.001	0.190	−0.30
	CG	7.49 ± 0.56	7.47 ± 0.57	0.132		
Standing long jump (cm)	EG	225.67 ± 16.33	235.20 ± 15.65	< 0.001	< 0.001	1.04
	CG	220.70 ± 17.15	223.80 ± 17.65	0.002		
Pull-ups (reps)	EG	5.50 ± 1.72	9.60 ± 1.81	< 0.001	< 0.001	2.03
	CG	5.13 ± 1.83	5.97 ± 2.03	< 0.001		
1,000-m run (s)	EG	257.49 ± 17.87	250.57 ± 18.05	< 0.001	0.597	−0.69
	CG	254.11 ± 19.54	253.15 ± 19.60	< 0.001		

EG, experimental group (blended learning); CG, control group (traditional learning). Within-group *p*-values are from paired *t*-tests; between-group *p*-values are from independent *t*-tests on post-test scores. Significant values (*p* < 0.05) are bold. Cohen's d was calculated for between-group comparisons as (M_EG - M_CG)/pooled SD at post-test. Within-group effect sizes are reported in the text.

^a^p-value for within-group comparison (pre-test vs. post-test).

^b^p-value for between-group comparison (post-test only).

^c^Cohen's d effect size for between-group comparison, calculated using the pooled post-test standard deviation.

Specifically, regarding the time effect, after the 12-week intervention, the traditional basketball learning model demonstrated only minor improvements in all tested physical fitness indicators. In contrast, the blended basketball learning model showed no significant improvement in endurance (1,000-m run), produced a moderate effect on lung capacity and explosive power (standing long jump), yielded a large effect on strength-endurance (pull-ups), and resulted in negative effects on speed (50-m run) and BMI.

The negative effect sizes observed for BMI (d = −0.54) and 50-meter sprint (d = −0.30) indicate that the experimental group showed less favorable changes on these measures compared to the control group. These findings may reflect the reduced emphasis on continuous hard running in the blended approach, which allocated more class time to skill practice and individualized feedback. A more detailed interpretation of these unexpected results is provided in the Discussion.

### 3.2 Basketball skills outcomes

With respect to basketball skills, both groups showed significant improvements in the 10 free throw attempts and half-court dribble and shot tests. However, the improvements were more pronounced in the blended learning (experimental) group. For the 10 free throw attempts, the experimental group achieved a large effect size (d = 1.63), compared to a large but relatively smaller effect size (d = 1.25) in the control group. In the half-court dribble and shot test, the experimental group again showed a large effect (d = 1.55), whereas the control group only reached a small effect (d = 0.41). These results indicate that the blended learning model is more effective than the traditional approach in enhancing basketball skills (see Table 6).

**Table 6:** Basketball skills outcomes.

Test	Group	Pre-test (M ±SD)	Post-test (M ±SD)	Within-group *p*^a^	Between-group *p*^b^ (post)	Cohen's d^c^ (between)
Set shot (successful shots out of 10)	EG	3.23 ± 0.86	6.83 ± 1.12	< 0.001	< 0.001	1.63
	CG	2.93 ± 1.11	4.73 ± 1.44	< 0.001		1.25
Half-court dribbling and shooting (s)	EG	28.63 ± 4.75	36.27 ± 4.91	< 0.001	< 0.001	1.55
	CG	27.73 ± 4.82	29.93 ± 5.43	< 0.001		0.41

Cohen's d^c^ calculated using pooled post-test SD. For the half-court test, lower times indicate better performance (faster completion).

^a^p-value for within-group comparison (pre-test vs. post-test).

^b^p-value for between-group comparison (post-test only).

^c^Cohen's d effect size for between-group comparison, calculated using the pooled post-test standard deviation.

## 4 Discussion

The study found that following the 12-week blended learning intervention, male students demonstrated significant improvements across several physical fitness measures, including BMI, lung capacity, pull-ups, standing long jump, 50-meter run, and 1,000-meter run. These findings align with previous studies that have reported significant improvements in BMI (Wang et al., 2022), lung capacity (Cordeiro et al., 2021), pull-up performance (Tao et al., 2024), standing long jump (Kyriakidis et al., 2022), 50-meter sprint (Zhao et al., 2025), and 1000-meter run (Wang et al., 2024a).

The post-test results revealed clear differences between the experimental and control groups in lung capacity, pull-ups, and standing long jump. These findings are consistent with previous research on blended learning in physical education. For example, one study involving sixth-grade students reported that a 14-week blended learning intervention led to improved fitness scores (Al Qudah et al., 2018). Similarly, another study indicated that blended learning contributed to greater gains in speed, strength, coordination, endurance, and flexibility than conventional instruction (Wang et al., 2022). Another study observed overall improvements in students' exercise attitudes following a 16-week blended learning program (Wang et al., 2024b).

However, not all studies are consistent; some have produced divergent results for measures such as the 50-meter run (Fadil and Pelana, 2023). Owing to variations in intervention duration, participant characteristics, and assessment methods, it remains difficult to draw definitive conclusions regarding the effectiveness of blended learning across all dimensions of physical fitness. Therefore, further research is needed to compare the impacts of blended and traditional teaching approaches in physical education.

In the 1,000-meter run, no significant difference in effectiveness was observed between blended learning and traditional teaching approaches, a result consistent with four previous studies (Wang et al., 2024a; Cui, 2023; Supriadi et al., 2023; Xu et al., 2023). Muscular endurance is defined as the ability of a muscle or muscle group to perform repeated contractions over an extended period under resistance (Anderson and Kearney, 1982). The absence of significant differences between the two groups may be attributed to the fact that improving muscular endurance requires specific load and intensity stimulation, which gradually enables the muscles to adapt to external resistance (Nygård et al., 1988; Miyamoto et al., 2016). However, a frequency of only one physical education session per week, whether using a blended or traditional teaching approach, is insufficient to provide the physiological stimulation needed to effectively induce such adaptive changes. Furthermore, students' muscular endurance showed limited improvement due to inadequate coordination between the training load in each class and the subsequent recovery. This indicates that relying solely on the existing instructional arrangement is unlikely to achieve the desired physical fitness outcomes and that curriculum design may need to be revised to increase training frequency and optimize load management.

In addition, this study shows that both blended learning and traditional teaching methods can effectively improve students' basketball skills, with blended learning having a more noticeable effect. For example, a study by Ding and Zhai on 90 male university students found that compared with the traditional teaching model (Ding and Zhai, 2023), the blended teaching model demonstrated significant advantages in helping students master basketball skills and enhancing their learning motivation. Similar positive effects of blended learning have also been observed in other sports such as soccer, volleyball, badminton, and dance (Bayyat, 2020; Haweni et al., 2025; Al-Hadidi, 2013; Zhu and Zhang, 2021).

The reason the experimental group got better at learning skills and building strength can be explained by two theories that work together. First, Self-Regulated Learning (SRL) theory posits that learners achieve better outcomes when they actively manage their own learning processes (Panadero, 2017; Zimmerman, 1990). The blended approach helped students take charge of their own learning. For example, they could choose when and what to watch before class, get quick feedback from online quizzes, and reflect on their progress by submitting videos after class. Giving students this kind of control may have helped them better understand how they learn and stay motivated, which in turn led to more effective practice and helped them remember what they learned for longer (Luo and Zhou, 2024). This self-regulated cycle of planning, monitoring, and reflection is central to the observed improvements in skill acquisition and muscular endurance.

Second, Vygotsky's sociocultural theory, particularly the concept of the zone of proximal development (ZPD), offers insight into the role of scaffolding in blended learning (Eun, 2019). By moving basic knowledge learning online to online platforms, in-class time could be dedicated to tasks within each student's ZPD, with the teacher providing individualized guidance and peer interactions fostering learn together and help each other learning (Chaiklin, 2003; Shabani et al., 2010). The combination of autonomous online preparation and socially mediated in-class practice likely optimized the learning environment for both skill development and physical conditioning.

The negative effect sizes observed for BMI and 50-meter sprint warrant careful interpretation. Regarding BMI, the slight decrease in the experimental group (d = −0.54) may reflect a reduction in body fat but given the concurrent large improvements in pull-ups and standing long jumping, it is plausible that muscle mass increased, which could have offset fat loss and resulted in only a small net change in BMI. This interpretation is consistent with previous research indicating that BMI is an imperfect proxy for body composition in physically active populations (Nevill et al., 2006).

These results are promising, but a few limitations need to be kept in mind. The sample size was relatively small, and all participants came from just one university. That means the findings may not apply to other groups or settings. Also, the 12-week program may not be long enough to see clear changes in aerobic endurance, as we saw with the 1,000-meter run results. In addition, we didn't use direct measures of body composition like DXA, so we can't draw firm conclusions about the BMI changes. Future research should include larger and more diverse groups of students, follow them for a longer period, and use more precise tools to measure body composition. This would help confirm and build on what we found.

## 5 Conclusion

In conclusion, this study demonstrated that blended learning effectively enhances male university students' physical fitness and basketball skills, particularly in muscular endurance and explosive power. By providing a more engaging, flexible, and efficient learning experience, blended learning positively contributes to physical education. However, the approach showed limited effects on BMI and sprint performance, suggesting that blended learning may require enhanced high-intensity training components to address these fitness dimensions. These findings have meaningful implications for the design and implementation of university physical education programs.

## Data Availability

The original contributions presented in the study are included in the article/supplementary material, further inquiries can be directed to the corresponding author.
